# Physiological and communicative emotional disconcordance in children on the autism spectrum

**DOI:** 10.1186/s11689-024-09567-4

**Published:** 2024-09-04

**Authors:** Emma Finkel, Eric Sah, McKenna Spaulding, John D. Herrington, Liza Tomczuk, Aaron Masino, Xueqin Pang, Anushua Bhattacharya, Darren Hedley, Yelena Kushleyeva, Phoebe Thomson, Natalie Doppelt, Jessica Tan, Jeffrey Pennington, Cheryl Dissanayake, Christopher P. Bonafide, Heather J. Nuske

**Affiliations:** 1 Center for Autism Research, Children’s Hospital of Philadelphia, Philadelphia, PA USA; 2 Sidney Kimmel Medical College, Thomas Jefferson University, Philadelphia, PA USA; 3 Penn Center for Mental Health, University of Pennsylvania, 3535 Market Street, Philadelphia, PA 19146 USA; 4 Department of Anesthesiology and Critical Care, Perelman School of Medicine, University of Pennsylvania, Philadelphia, PA USA; 5 Department of Biomedical and Health Informatics, Children’s Hospital of Philadelphia, Philadelphia, PA USA; 6 Olga Tennison Autism Research Centre, La Trobe University, Melbourne, VIC Australia; 7 Autism Center, Child Mind Institute, New York, NY USA; 8 Department of Biomedical and Health Informatics, Children’s Hospital of Philadelphia, Philadelphia, PA USA; 9 Division of General Pediatrics, Children’s Hospital of Philadelphia, Philadelphia, PA USA

**Keywords:** Emotional concordance, Emotional coherence, Autism, Heart rate, Stress communication

## Abstract

**Background:**

Individuals on the autism spectrum commonly have differences from non-autistic people in expressing their emotions using communicative behaviors, such as facial expressions. However, it is not yet clear if this reduced expressivity stems from reduced physiological reactivity in emotional contexts or if individuals react internally, but do not show these reactions externally to others. We hypothesized that autism is characterized by a discordance between in-the-moment internal psychophysiological arousal and external communicative expressions of emotion.

**Methods:**

Forty-one children on the autism spectrum and 39 non-autistic, typically developing (TD) children of two age groups (2–4 and 8–12 years) participated in a low-level stress task whilst wearing a wireless electrocardiogram. Children’s negative emotional expressions (facial, vocal, bodily) were coded following standardized protocols. Alexithymia traits were assessed using the Children’s Alexithymia Measure with school-aged children only. Data analyses involved ANOVAs, correlations, and sensitivity analyses.

**Results:**

There were no group differences in physiological arousal (heart rate) or in communicative expressions of stress to the stress task. For TD preschoolers, physiological arousal during the stress task was associated with vocal expressions and for TD school-aged children, they were associated with facial and bodily expressions. By contrast, for children on the autism spectrum, physiological arousal during the stress tasks was not associated with communicative expressions across age groups.

**Conclusions:**

Our findings suggest that children on the autism spectrum might experience emotional disconcordance, in that their physiological arousal does not align with their communicative expressions. Therefore, the internally experienced stress of children on the autism spectrum may be inadvertently missed by teachers and caregivers and, consequently, learning opportunities for teaching emotional communication and regulation may be also missed. Our results support the use of wearable biosensors to facilitate such interventions in children on the autism spectrum.

**Supplementary Information:**

The online version contains supplementary material available at 10.1186/s11689-024-09567-4.

The ability to communicate emotions is integral to healthy psycho-social development [101]. Beginning in infancy, children use communicative expressions to signal distress to parents, creating emotion-regulation learning opportunities whereby parents model and shape effective emotion communication and regulation [25, 70, 92, 109]. Children’s learning of appropriate ways to verbally and nonverbally express their negative emotions through interactions also facilitates the development of social relationships [35] and can prevent escalation to anxiety, aggression, or associated psychopathology [5, 10, 16, 44, 47, 62, 68, 70, 88]. Our use of the term “negative emotions” refers to the valence of emotions. Given the functional utility and importance of negative emotions [75], negative emotions were studied as they are critical for emotional well-being and psychological health [14, 76].

Children on the autism spectrum who are characterized by differences in social communication and interaction as well as restricted and repetitive behaviors and interests [1], exhibit difficulties expressing emotions using clear, unambiguous communicative behaviors (for review, see [71]). Individuals on the autism spectrum, relative to non-autistic, typically developing (TD) peers, may also present with an absence of or reduced emotional expression, such as decreased positive affect, or flat/neutral affect shown by facial expressions [46, 71, 94, 108]. We note however that autism is a complex, nuanced condition with much heterogeneity in autism symptom presentation, and not all individuals on the autism spectrum experience these symptoms [26, 71]. Reduced ability to express negative emotions, such as stress, using typical communication channels may contribute to emotion regulation difficulties downstream. Indeed, many children on the autism spectrum release tension in undesirable ways, for example, via challenging behaviors, such as aggression and self-injury [39, 59, 63, 65].

Communicative expression of emotions is just one of many components of an affective response. According to many models of affective responses, such as the biopsychosocial model of challenge and threat [8], an individual’s affective responses include multiple components (e.g., behaviors/expressions, subjective experiences/cognitive appraisals, and physiological reactivity). Physiological reactivity, such as brain activity, skin conductance, pupil dilation and heart rate, can indicate affective arousal. The advantage of heart rate measurement over other indices is that wearable heart rate devices can be used during everyday activities and reliably so during non-vigorous movement [69, 74, 104, 107]. Measured in beats per minute (BPM), heart rate has long been used as an index of affective arousal during emotional distress tasks, including in children on the autism spectrum, and will therefore be the index for physiological reactivity in this study [2, 9, 34, 100, 103]. Physiological measures are useful to record affective arousal during emotional distress tasks in children on the autism spectrum who have difficulties with emotional expression, complementing self-report and visual observation. Therefore, physiology may reveal moments of affective arousal not shown by other emotional channels in children on the autism spectrum [34, 65, 106].

Multiple studies have found that individuals on the autism spectrum, relative to TD controls, have decreased heart rate variability at baseline, supporting the idea that autism is associated with dysregulation of heart rate (e.g., [2, 34, 103]). Research assessing changes in heart rate from baseline during stress-inducing tasks has shows increased heart rate increases during low-level stress tasks in both TD and autistic individuals (e.g., [90, 103]), though studies show inconsistency in magnitude of this response in individuals on the autism spectrum relative to controls (for review, see [56]). Most studies used stress-inducing tasks that do not involve sensory (e.g., blood draw) or social stimuli (e.g., Trier Social Stress Test). There have been a limited number of studies that have employed psychosocial tasks such as the one used in the current study, the Transparent Box task (see *Methods* section for details), which mimics a real-life situation involving children waiting to play with toys. Studies that have used psychosocial tasks demonstrate similar physiological response to stress between TD and autistic individuals, with significant individual variability in the autism group [30, 32].

## Emotional concordance: coordination between physiological and communicative reactivity

Emotional concordance, also referred to as emotional coherence or emotional congruence, is the coordination between behavioral expression, subjective experience, and physiological arousal [38]. The concept of emotional concordance emerged from traditional emotion theories (James-Lange, Cannon-Bard, and Schachter-Singer) from the notion that affective responses involve some level of coordination across these response systems [38, 51, 73]. Emotional concordance implies that physiological changes are accompanied by analogous communicative behavioral expressions and subjective experience. For example, the emotion of anger would include an increased heart rate (physiological response), furrowing of eyebrows (communicative behavior), and thoughts of fury toward another (subjective experience). Emotional discordance, in contrast, occurs when there is a lack of association between the three emotional response systems [38]. For example, discordance would occur if there were an increase in heart rate but no accompanying communicative or behavioral expression of anger. Rather than being the norm, Lang [51] argued that emotional concordance varies among individuals. Some studies show strong correlations between behavioral expression and subjective experience in TD individuals (i.e., *r* = 0.74), but only moderate correlations between behavioral expressions and physiological arousal (i.e., *r* = -0.52) [64]. However, evidence of concordance in the TD population remains limited and inconsistent [38, 52]. Early emotion theories and modern psychological constructionist hypothesis suggest appraisal or interoception of physiological arousal is needed prior to conscious feeling of emotion [3, 4, 53, 58, 87]. Moreover, conscious feeling may be a necessary precursor to intentional outward expression or communication of emotions to others as opposed to automatic bodily responses [11]. Therefore, studying emotional coherence in populations with compromised interoceptive awareness, such as those with alexithymia, is important to understanding the link between appraisal and emotional expression. Alexithymia refers to the inability in perception and verbalization of one’s emotions as well as externally-oriented thinking and is about 50% prevalent in individuals on the autism spectrum compared to about 5% in general population [50, 93]. Those with alexithymia commonly show difficulty detecting and regulating bodily processes. Therefore, more research is needed on emotional concordance, both in typical and atypical child populations.

Few studies have empirically examined emotional concordance in autism. Providing seminal evidence of emotional discordance in autism, Costa et al., [16] found that, relative to TD children, children on the autism spectrum presented with a greater mismatch between facial expression and bodily expression during distressing situations.Children on the autism spectrum have stronger traits of alexithymia [16, 31, 77, 86] found that alexithymia traits accounted for emotional discordance in their sample as well. Keith et al. [48] reported concordance between self-reported, but not parent-reported, anxiety and autonomic arousal in adolescents on the autism spectrum with at least 80 IQ based on Wechsler scale. An unanswered question, however, is whether there is emotional discordance in autism between internal physiological versus external communicative emotional reactions. While children on the autism spectrum exhibit typical physiological distress responses (e.g., [90, 103]), they have difficulties communicating emotions [46, 71]. This suggests reduced emotional concordance compared to TD children. However, research has yet to investigate this question.

## The current study

The aim in the current study was to address these research gaps by investigating internal physiological and external communicative emotional concordance in TD children and children on the autism spectrum. Based on the literature, four hypotheses were developed regarding responses during a low-level stress task relative to a resting-state task:

1. Both groups will have increased physiological response (i.e., heart rate increase)
2. Relative to TD children, children on the autism spectrum will exhibit fewer communicative behaviors (i.e., facial, vocal, and bodily reactions of fear, sadness or anger)
3. Relative to TD children, children on the autism spectrum will have less emotional concordance between physiological and communicative reactivity
4. Alexithymia (assessed via parent report) will account for emotional discordance (i.e., less emotional concordance) in the children on the autism spectrum

## Methods

### Participants

Children were recruited across two age groups for this study: preschoolers (2–4 years) and school-aged children (8–12 years). The preschooler sample included 20 children on the autism spectrum and 20 typically developing children, and the school-age sample included 21 children on the autism spectrum and 19 typically developing children. The chosen age groups were selected to investigate emotional concordance in both preschoolers and school-aged children, as previous research primarily investigated emotional concordance in individuals on the autism spectrum has been conducted on adolescents and adults [24, 48, 72]. Importantly, neurotypical adolescents and adults can be quite adept at managing their emotions by masking their emotional expressions [81, 84], and this allowed us to explore emotional concordance without the cofounding factor of emotional masking behaviors. The groups were separated to determine developmental changes in emotional expressions and to ensure appropriate use of the *Children’s Alexithymia Measure*, which is validated only for use with children who are in our school-aged children group. All children were recruited as part of larger studies (see Procedures). Given the novelty of the methods applied to children on the autism spectrum, sample size was based on recruitment rather than a formal power analysis. Therefore, the study was underpowered (based on the observed minimum effect size in this study, 50 participants per group would have been needed). Diagnoses of autism were confirmed with the Autism Diagnostic Observation Schedule, second edition (ADOS-2; [54]). ADOS-2 calibrated Comparison Scores (CS) are provided (see Table 1) on a 10-point scale with scores anchored to ADOS-2 classifications, based on the raw overall scores, module used, and age of the child. Severity scores are provided by the publisher for Modules 1–3 [54]. For the preschool sample, CS for the toddler module were based on algorithms provided by Esler et al. ([21],see also [37]). Five children recruited into the autism group scored below threshold, and two children recruited into the TD group scored above threshold on the ADOS,all were excluded from the analyses (not included in the total participants reported above).

**Table 1:** Participant characteristics

	**TD**	**ASD**	**Comparison**
*Preschool*			
Age: M (SD)	3.25 (0.85)	3.46 (0.54)	*p* = .35
Sex (M:F)	17:3	13:7	*p* = .27
IQ (MSEL)	110.05 (17.35)	80.25 (26.18)	*p* < .001*
Autism severity (ADOS)	-	6.85 (2.62)	-
*School-age*			
Age: M (SD)	10.00 (1.29)	9.67 (1.39)	*p* = .44
Sex (M:F)	13:6	18:4	*p* = .47
IQ (SB)	104.11 (11.67)	89.90 (20.54)	*p* = .01*
Autism severity (ADOS)	1.26 (0.65)	6.95 (2.21)	*p* < .001*
Alexithymia (CAM)	5.63 (7.28)	15.09 (8.79)	*p* < .001*

*ADOS* Autism Diagnostic Observation Schedule Comparison Score, *CAM* Children’s Alexithymia Measure Total Score, *MSEL* Mullen Scales of Early Learning Standard Score, *SB* Stanford Binet Abbreviated Battery IQ Standard Score

For the preschool age sample, a developmental quotient (DQ) was obtained using the Mullen Scales of Early Learning [66], and for the school-age sample, IQ was assessed through the Stanford-Binet Intelligence Test from Stanford-Binet Intelligence Scales, Fifth Edition [80] Abbreviated Battery IQ scales (ABIQ). For the school-age sample, two children recruited into the TD group that had below average (< 85) IQs were excluded in the analysis so that typical emotional concordance could be examined (not included in the total participants reported above). Following the recommendations of Dykens and Lense [18], children on the autism spectrum with below average (< 85) DQ or IQ were retained in the sample due to the high prevalence of cognitive and language delays/impairments in autism. Both the preschooler and school-age samples included children on the autism spectrum with a wide range of standard score [45], with 50% and 16%, respectively, with standard score < 70, 10% and 16%, respectively, with standard score 70–84, and 40% and 68%, respectively, with standard score ≥ 85. The IQ ranges for each sub-group were as follows: preschool TD: 85–132, preschool autism: 32–120, school-age TD: 85–121, school-age autism: 47–121. The diagnostic groups (autism, TD) within each age group were matched on age and sex. See Table 1 for participant characteristics and group comparison statistics.

### Experimental tasks

#### Low-level stress task

We used the Transparent Box task from the Preschool and Middle Childhood versions of the Laboratory Temperament Assessment Battery (Lab-TAB; [28, 29]) as a low-level stress task as it was designed to induce frustration and mild distress in children. The child is given a transparent box filled with attractive toys that the child cannot access for a period of time. In the preschool version, the child is asked to choose which toy (of two) he/she would like to play with, is given a container with the toy inside and then the experimenter leaves the room. Toys are in a transparent container, which children this age cannot open (wide cylinder container shut with screw on lid closed extra tight). In the middle childhood version, the child is given a transparent box of attractive toys and the wrong set of keys, and then the experimenter leaves the room. After a set amount of time (2 min for the preschool version, 4 min for the school-age version), the experimenter apologizes (for not opening the container for preschoolers, for the “mix-up” with the keys for the older children) and either opens the container for the child or shows them how to do it themselves. If the child somehow opened the container beforehand, the task is finished (three preschool children and one school-aged child opened the container before the task was over and were excluded from the study (and not counted in Table 1)). As a positive resolution to the task, the experimenter joins in play with the child with the attractive toys or lets him/her play alone if preferred. Lab-TAB tasks have been validated as effective in inducing negative emotions in children (e.g., [57, 67, 70]).

#### Resting state period

To establish a baseline heart rate for each child, data were collected during a resting state period, when the child was calm (i.e., no negative emotion or high intensity positive emotion), not moving too much (three-coordinate accelerometer and video data were reviewed and data streams were referenced to ensure data did not have movement artifacts) and not interacting with others. In the preschool sample, for 35 children this was during watching a relaxing, developmentally appropriate video (the last 3 min of *Sweet Dreams Spot* [15]), for five children (all children on the autism spectrum), this was when they were engaged in free play while sitting. The heart rate during resting periods of the five preschoolers on the autism spectrum that were captured during free play while sitting were no different from the heart rate during resting periods of the 15 preschoolers on the autism spectrum that were captured during the relaxing video (*t*(18) = -0.52, *p* = 0.61). A 3-min resting state period was selected given the age of children, and used previously as an alternative to 5-min epochs in younger samples [15]. For the school-age sample, the resting state period was while children watched a relaxing video on a computer that was originally designed for fMRI resting-state (*Inscapes*,[102]). All school-aged children’s baselines were measured during this video, with the first 5 min of the video used as the baseline period.

### Physiological data

#### Heart rate devices

In the preschooler study, children wore vests with sewn-in pockets to house the Biopac BioNomadix ECG and accelerometer module transmitters. ECG electrodes which connected to the transmitters were placed on children’s back (instead of chest) to avoid children pulling at them (e.g., [68]). Transmitters connected wirelessly to the Biopac MP150 receiver, allowing children free movement around the testing room. This system sampled at 1 kHz and no sampling filters were used during recording.

In the school-age study, children wore five consumer-grade devices that recorded physiological responses throughout the tasks, as part of a larger study on wearable devices. For the purposes of the current study, data collected from the Polar H7 ECG chest strap, and if not available, from the Mio Fuse photoplethysmography wristband, were used for analysis, as we and others have found these devices to be valid and reliable devices compared to traditional wired ECG [69, 74, 104, 107]. Data were collected from the Polar H7 if the device had collected at least 80% of the data for the task, otherwise the Mio Fuse was used. The Polar H7 was used for 75% of the participants. This system sampled at 1 kHz and proprietary sampling filtering algorithms embedded in the devices’ microcontrollers, which detect when heartbeats occur, broadcasted directly via Bluetooth Low Energy the inter-beat intervals to the recording computer.

#### Heart rate event marking, cleaning and extraction

For the preschool sample, offline event marking was used whereby, first, the timing of the low-level stress task and resting state periods were labelled on the software using the synced video stream. ECG data were processed in Biopac AcqKnowledge version 4.4.2. Second, a band-pass (Finite Impulse Response) filter was applied to de-noise the ECG signal, with low frequency fixed at 1 Hz and high frequency at 35 Hz (e.g., [49, 82]). Third, QRS complexes were labelled and visually inspected for artifacts in the marked data sections [99]. In patches of data artifacts, due to movement (accelerometer data were referenced), data were either linearly interpolated in-between R peaks and/or R signals were amplified, so that extracted data metrics were unaffected by artifacts (Task Force of the ESC and [99]). We binned analyses into 30-s epochs, and if more than 5 secs needed to be interpolated, that epoch was excluded from the analyses. Based on these criteria, 7 rest epochs and 5 stress epochs of 5 autism participants were deleted. In both groups, the total amount of data interpolated was similar across the rest (TD: *M* = 3.85 secs, *SD* = 4.20 secs; autism: *M* = 8.64 secs, *SD* = 9.71 secs) and stress (TD: *M* = 3.59 secs, *SD* = 2.81 secs; autism: *M* = 8.19 secs, *SD* = 8.40 secs) conditions. For the school-age sample, live event marking was used whereby research assistants watching the session live from a clinical observation room marked the start and end of the low-level stress task and resting state periods on custom-built software created for the project (as described in [61]). The heart rate data collected by the wearable devices were inputted into Kubios HRV software [98]. On Kubios software, if an RR (time in between heart-beat) interval deviates from a median-filtered local average by more than a specific threshold (very low: 0.45 s to very strong: 0.05 s), it is marked for corrections (https://www.kubios.com/blog/preprocessing-of-hrv-data/). A strong threshold filter (0.15 s) was used to correct for artifacts as it was the lowest possible correlation level that effectively corrects abnormal beats without causing overcorrection. For all children, mean heart rate and peak heart rate were extracted from the software for the low-level stress task and resting state periods.

### Communicative expressions behavioral coding

A behavioral coding manual was created based on the codes provided in the respective Lab-TAB manuals and the AFFEX Facial Expression Reference Chart [29, 42]. Codes included negative facial and bodily reactions matched to fear, sadness, or anger (a composite of the three emotions comprised the final codes), and negative vocalizations matched to levels of distress. For each, codes ranged from 0 = no expression to 3 = extreme expression, and further specification was given for each communication channel. Facial expression codes referenced to specific facial muscle movements as per the AFFEX Facial Expression Reference Chart, whereby a code of 1 = one facial area involved, 2 = two facial areas involved and 3 = three facial areas involved.

Vocal expression codes differed by intensity (1 = mild, 2 = moderate, 3 = extreme), and coders calibrated to the lead coder on these levels during coding training. Given the wide range of verbal abilities in children on the autism spectrum, ranging from minimally verbal to typically fluent children, vocal codes incorporated sounds (e.g., whines), affective prosody (i.e., pitch, loudness, timbre, speech rate, and pauses), as well as words or content (i.e., meaning of words indicating distress).

Bodily expression codes referenced specific behaviors, for instance with anger: 1 = slight bodily tensing or mild frustration behavior (e.g., sighing with frustration, placing hand/arm on table with more force than necessary); 2 = moderate bodily tensing (e.g., balling the fists) or moderate frustration behavior (e.g., pushing away from the table, sighing heavily, pushing the box away, slamming hand/arm on table with definite intention); 3 = high bodily tensing (e.g., trembling) or high frustration behavior (e.g., throwing the container or keys). The full coding protocol and calibration procedure are available on request.

For both samples, coders blind to diagnostic group and study hypotheses coded behaviors, after group training on the codes and achieving inter-rater reliability (IRR) of 80% or higher on three consecutive training videos with the lead coder. The lead coder also double-coded a minimum of 20% of each coder’s videos (Preschooler sample: IRR *M* = 0.81, *SD* = 0.05, *Range* = 0.71–0.89; School-age sample: IRR *M* = 0.86, *SD* = 0.07, *Range* = 0.78–1.00) to check ongoing reliability maintenance. Any ambiguous child behaviors were discussed in order to reach a consensus. For the preschooler sample, manual coding sheets were used. For the school-age sample, coders used the Behavioral Observation Research Interactive Software (BORIS) to code behaviors [22].

### Alexithymia

In order to examine alexithymia traits in the children, we used the Children’s Alexithymia Measure (CAM; [105]), a parent/caregiver report measure that measures alexithymia as a unidimensional factor (difficulties expressing feelings). It is appropriate for children aged 5–17 and was therefore only used with the older school-age sample. As no reliable measures of alexithymia are available for preschool age children, no alexithymia measure was used in the younger sample. The CAM produces a total score, with higher scores indicating higher alexithymia traits. The internal consistency for each diagnostic group was moderate to high in this sample (autism: Cronbach’s *α* = 0.92; TD: Cronbach’s *α* = 0.68).

### Procedure

Testing was completed at the Olga Tennison Autism Research Center, La Trobe University for the preschool sample, and at the Center for Autism Research, Children’s Hospital of Philadelphia for the school-age sample following common procedures. For both sites, after review of study procedures including reviewing a picture book of tasks included in the study, parents provided their informed consent to participate in the study. For those able, child participants in the school-age sample were invited to give assent to participate. Consent/assent was obtained according to the Declaration of Helsinki (BMJ 1991; 302: 1194). Parents were asked to assist with putting on the devices to record physiological response and were then provided with a laptop or seated at a computer to complete the study questionnaires during the session. The research was approved by the La Trobe University Human Ethics Committee for the preschool sample and the University of Pennsylvania and Children’s Hospital of Philadelphia for the school-age sample.

### Data analysis

Data were first analyzed for outliers and normality. As heart rate variables in both samples and communication variables in the school-age sample were non-normally distributed, these variables were log-transformed. As the groups differed on cognitive ability (DQ/IQ), zero-order correlations between cognitive ability and the variables of interest were explored. No variables were significantly associated with cognitive ability in any diagnostic or age group (autism preschool *p* range = 0.15—0.99; TD preschool* p* range = 0.26—0.69; autism school-age *p* range = 0.16—0.94; TD school-age* p* range = 0.31—0.84), so cognitive ability was not controlled for in subsequent analyses. See Supplementary Table 1 and Table 2 for all zero-order correlations examined for association with cognitive ability for the preschool group and the school-age group, respectively. To test Hypothesis 1 (that groups have comparable physiological stress responses), four 2-way (group [autism, TD] × task [rest, stress]) repeated measures ANOVAs were conducted; one on mean BPM and another on peak BPM during the task, relative to mean BPM at rest, for each child sample. To test Hypothesis 2 (that the autism group would express fewer communicative behaviors than the TD group), two 2-way (group [autism, TD] × communication channel [facial, vocal, bodily]) repeated measures ANOVAs were conducted, one for each child sample. Post-hoc pairwise comparisons were conducted.

The main hypothesis, Hypothesis 3 (that TD children, but not children on the autism spectrum, will show emotional concordance) was tested with two sets of analyses. First, we conducted machine learning Maximal Information Coefficient analyses [78] to determine the probability of bivariate associations. Maximal Information Coefficient analyses offer an advantage over standard inferential statistics/hypothesis testing when sample sizes are very small because they are unconstrained by linear associations and are thus more equipped to capture the nuances of complex data [40, 79, 97]. However, Maximal Information Coefficients do not show directionality and are not subject to a significance level. Second, therefore, to confirm these findings, as sensitivity analyses, we also conducted follow-up partial Pearson’s correlations run between communicative expressions (facial, vocal, bodily) and heart rate (mean and peak BPM) during the stress task, controlling for heart rate (mean BPM) at rest, to examine if variation in analytic practice affected results [36]. A False Discovery Rate correction [7] was applied to the results to control for multiple testing. As PPG-based heart rate data and ECG-based heart rate data may not be equivalent, to test if results were affected by incorporation of PPG as well as ECG data in the school-age group, we conducted partial correlation sensitivity analyses excluding PPG data (with data from 3 TD children and 3 children on the autism spectrum excluded).

To test Hypothesis 4 (that alexithymia accounts for emotional discordance in autism), partial correlations were run for the school-aged sample between alexithymia (CAM total score) and computed heart rate (mean and peak BPM) during the stress task × communicative expressions (facial, vocal, bodily) interaction terms, controlling for heart rate (mean BPM) at rest. As additional sensitivity analyses to examine consistency of findings, throughout each of the above analyses we present both mean and peak BPM during the stress task relative to mean BPM at rest as the decision to use one or the other may have affected results and therefore interpretation [96]. All statistical analyses including ANOVA, correlations, and sensitivity were completed in SPSS.

## Results

The mean age of preschool TD group and the autism group were 3.25 ± 0.85 and 3.46 ± 0.54. The mean IQ were 110.05 ± 17.35 and 80.25 ± 26.18 for preschool TD group and the autism group respectively. The mean age of school-age TD group and the autism group were 10.00 ± 1.29 and 9.67 ± 1.39. The mean IQ were 104.11 ± 11.67 and 89.90 ± 20.54 for school-age TD group and the autism group respectively. For preschool autism group, the autism severity score was 6.85 ± 2.62. For school-age autism group, the autism severity score was 6.95 ± 2.21. Full participant characteristics are shown in Table 1.

For Hypotheses 1 and 2, eta-squared (*η*^*2*^) effect sizes are provided for each ANOVA main and interaction effect reported below. For the purposes of the study, we followed Cohen’s [13] guidelines for interpreting effect sizes, whereby 0.01 = small, 0.06 = medium and 0.14 = a large effect. For Hypotheses 3 and 4, Pearson correlation coefficients are provided, whereby 0.10 = small, 0.30 = medium and 0.50 = a large effect [13].

### Hypothesis 1: physiological reactivity

**Fig. 1:**
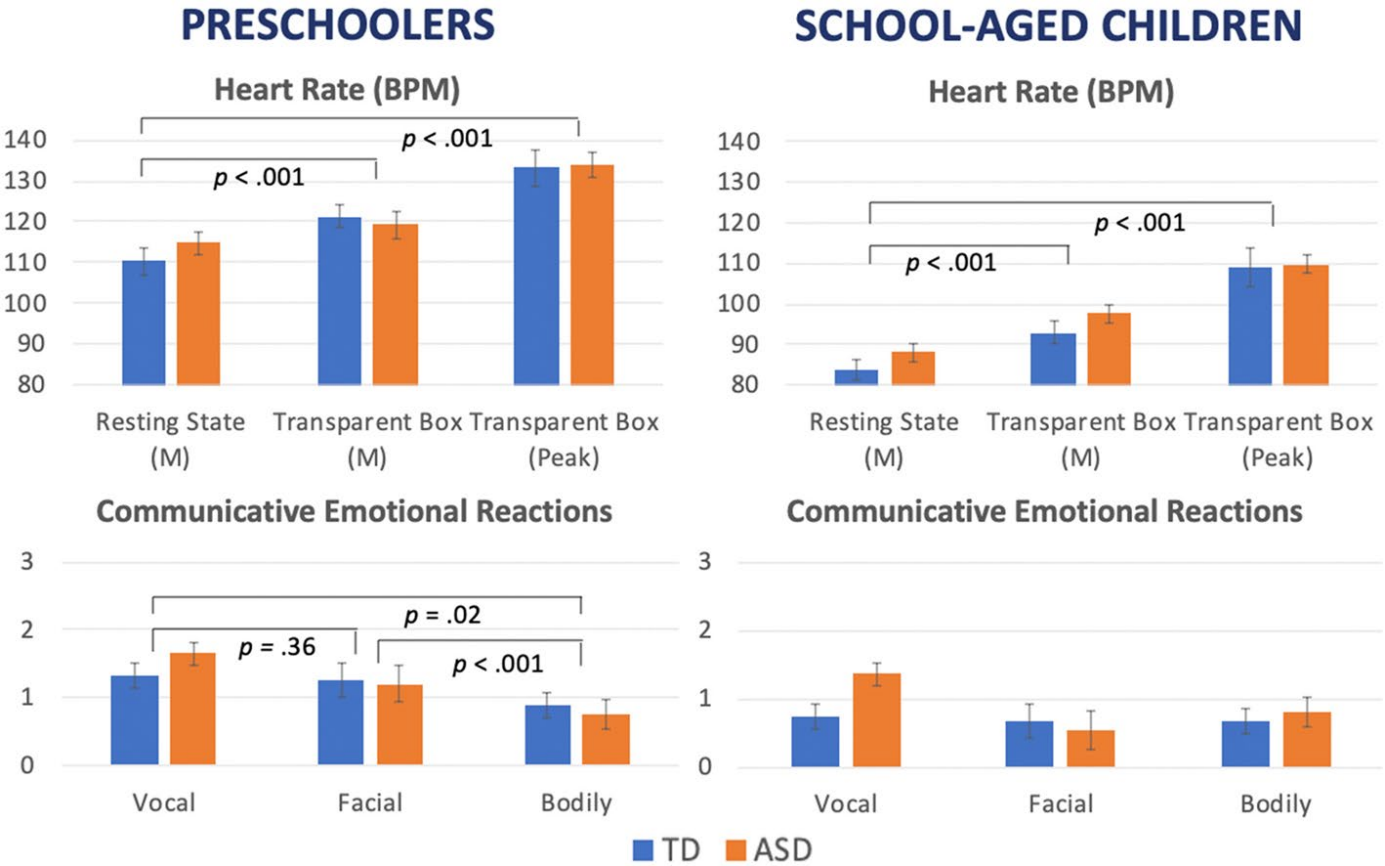
Physiological and communicative reactivity in each diagnostic and age group. *Note. p* < .05*;* p* < .01**; *p* < .001***

We first checked for any difference in resting state heart rate; no diagnostic group differences were found for either the preschool, *t*(38) = -1.10, *p* = 0.28, or the school-age group, *t*(38) = -1.23, *p* = 0.23. Regarding physiological responses to the stress task, for preschoolers and school-aged children, heart rate (BPM) was higher in the task compared to resting state such that there was a significant main effect of Task for mean BPM, *F*(1,38) = 19.24, *p* < 0.001,* η*^*2*^ = 0.35 and *F*(1,38) = 55.29, *p* < 0.001,* η*^*2*^ = 0.59, respectively, and peak BPM, *F*(1,38) = 145.45, *p* < 0.001,* η*^*2*^ = 0.80 and *F*(1,38) = 108.18, *p* < 0.001,* η*^*2*^ = 0.74, respectively. The main effect of group was not significant at either age or heart rate variable (preschool mean: *F*(1,38) = 0.13, *p* = 0.72,* η*^*2*^ = 0.004; preschool peak: *F*(1,38) = 0.56, *p* = 0.46,* η*^*2*^ = 0.02; school-age mean: *F*(1,38) = 1.92, *p* = 0.17,* η*^*2*^ = 0.05; school-age peak: *F*(1,38) = 0.92, *p* = 0.34,* η*^*2*^ = 0.02). For the preschool group, there was a marginally significant task × group interaction effect for mean BPM, *F*(1,38) = 3.92, *p* = 0.055,* η*^*2*^ = 0.10. Pairwise comparisons showed that the difference between mean BPM in the rest and stress tasks was significant and large in the TD group, *p* < 0.001, and marginal in the autism group, *p* = 0.096. The task × group interaction effect for preschool peak BPM (*F*(1,38) = 1.14, *p* = 0.29,* η*^*2*^ = 0.03), school-age mean BPM (*F*(1,38) < 0.001, *p* = 0.99,* η*^*2*^ < 0.001), and school-age peak BPM (*F*(1,38) = 0.51, *p* = 0.48,* η*^*2*^ = 0.01) were all non-significant. Means are displayed in Fig. 1a.

### Hypothesis 2: communicative reactivity

For preschool aged children, the main effect of Communication channel was significant, *F*(2,38) = 10.70, *p* < 0.001,* η*^*2*^ = 0.37, with no main effect of Group (*F*(1,38) = 0.03, *p* = 0.87,* η*^*2*^ = 0.001) or interaction effect (*F*(2,37) = 1.40, *p* = 0.26,* η*^*2*^ = 0.07). Pairwise comparisons showed that facial and vocal reactions were higher than bodily reactions during the stress task, *p* = 0.02, and *p* < 0.001, respectively, with no differences between facial and vocal reactions, *p* = 0.36. For school-aged children, there were no Group (*F*(2,37) = 0.51, *p* = 0.48,* η*^*2*^ = 0.01), Communication channel (*F*(2,37) = 0.86, *p* = 0.43,* η*^*2*^ = 0.04) or interaction effects (*F*(2,37) = 0.68, *p* = 0.51,* η*^*2*^ = 0.04). Means are displayed in Fig. 1b.

### Hypothesis 3: emotional concordance

**Fig. 2:**
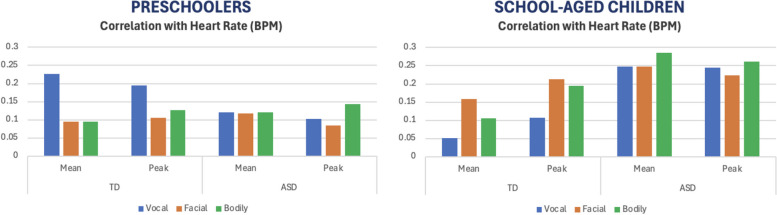
Maximal information coefficients between physiological and communicative reactivity in each group. Note. ASD = Autism Spectrum Disorder group; TD = Typically Developing group. Findings in the preschooler sample are consistent with the Pearson’s correlation analyses shown in Table 2 (emotion concordance with vocal expressions in TD, no emotional concordance in ASD). With the school-aged sample, findings in TD also are consistent with the Pearson’s correlation analyses shown in Table 2, and specify these reactions are driven by peak, and not mean heart rate. Findings of the ASD school-aged sample here reveal relationships between all expression types (vocal, facial, bodily) and both mean and peak heart rate, in contrast with the original analyses which only found significant emotion concordance with vocal expressions and peak heart rate. However, Maximal Information Coefficients do not show directionality and are not subject to a significance level. Taking this into account, therefore these findings are also consistent with the Pearson’s correlation analyses in terms of magnitude of associations, and the Pearson’s correlation analyses provides more cues to interpretation (directionality, significance level). In sum, this Maximal Information Coefficients analyses largely confirm the original analyses

The Maximal Information Coefficients findings are presented in Fig. 2. For preschoolers in the TD group but not the autism group there were associations between physiological and vocal reactivity. For school-aged children, by contrast, in the autism group, there were associations of physiological reactivity with all types of reactivity (vocal, facial and bodily), and in the TD group, there were associations of physiological with facial and bodily reactivity.

Follow-up Pearson’s correlation analyses shown in Table 2. These findings confirm the Maximal Information Coefficients findings in that in the preschool group, emotion concordance with vocal expressions was identified in TD but no emotional concordance was identified in autism. In the school-aged sample, findings in TD are also consistent with the previous analyses (although Maximal Information Coefficients findings more specifically suggest this finding is driven by peak, and not mean heart rate). Findings of the school-aged children in the autism group here specify that emotion concordance is with vocal expressions (here, associations with facial and bodily reactivity were not positive or did not approach significance). However, after applying the False Discovery Rate correction [7], the association between physiological and vocal reactivity in the school-age autism group no longer was significant. In the sensitivity analysis excluding PPG data from the school-age group, the pattern of findings was identical (see Supplementary Table 3). We also include raw analyses without any interpolation for comparison (Supplementary Table 4).

**Table 2:** Correlations between physiological and communicative reactivity in each group

	**Preschoolers**				**School-Aged Children**			
	TD		ASD		TD		ASD	
	*Heart Rate (BPM)*				*Heart Rate (BPM)*			
	M	Peak	M	Peak	M	Peak	M	Peak
*Vocal*	.48*	**.37#**	-.30	.22	.10	-.03	.28	**.32#**
*Facial*	.10	.14	-.19	.27	.43*	.53*	-.26	-.15
*Bodily*	-.08	-.11	-.11	.06	**.35#**	.58**	.23	.26

All correlations are partial, controlling for baseline physiology. *# p* < .10, * p < .05, ** p < *.01.* Findings that remain significant at *p* < .05 after False Discovery Rate correction applied [7] are underlined

### Hypothesis 4: alexithymia

For the school-aged children on the autism spectrum, alexithymia traits were related to the physiological and facial reactivity interaction term, whereas for the school-aged TD children, alexithymia traits were associated with the physiological and vocal reactivity interaction term and the physiological and bodily reactivity interaction term. Correlation coefficients are displayed in Supplementary Table 5.

## Discussion

In this study, we aimed to examine emotional concordance in TD children and children on the autism spectrum and its association with alexithymia. We found that preschoolers on the autism spectrum had no alignment between physiological and communicative reactions (i.e., they showed a pattern of emotional discordance). In the school-aged children on the autism spectrum, there was only marginal evidence of emotional concordance, which was similar to that found in younger (preschool-aged) TD children (emotional concordance with vocal reactivity). However, after correcting for multiple testing, this finding was no longer significant; confirmation is thus needed in a follow-up study. Together, these findings suggest that children on the autism spectrum appear to have disrupted and possibly delayed emotional concordance, potentially due to general impairments these children experience in using communicative behaviors like facial expression to convey their emotions to others [6, 65, 70, 71].

In line with these findings, we found that emotional concordance in autism was marginally related to alexithymia traits. This latter finding fits with the known challenges individuals on the autism spectrum have in perceiving their own emotions (e.g., [77]), and difficulties in physiological awareness or interoception [17, 24]. Indeed, interoception accuracy has been found to be associated with alexithymia in a study on adults on the autism spectrum [89]. Together these findings suggest that awareness of physiological responses may be key to emotional coherence, and that difficulties in this area underlie reduced emotional coherence in autism. Our findings suggest alexithymia and emotional expressions are associated and extend Schachter and Singer’s [87] seminal work on the relation between emotional appraisal of physiological arousal and conscious feeling. However, all findings with alexithymia were marginal and were not retained after applying the correction for multiple testing,therefore, further research is needed to verify any associations between alexithymia and emotional discordance in autism and to examine conscious feeling as a mediator between emotional appraisal of physiological arousal and emotional expression. Also, given that alexithymia measures have not yet been adapted for younger children, alexithymia was only investigated in half of the sample (the school-aged group), and should be followed up with a larger sample.

The findings are consistent with the idea that typically developing children first develop emotional concordance with vocal expressions, prior to facial and bodily expressions. These findings mirror the developmental sequelae of emotional expressivity throughout the first years of life whereby newborn infants first reactively express stress with crying before engaging the appropriate facial and bodily musculature to express emotions via those channels (e.g., [60]). Throughout early childhood, there is also the shift from automatic, non-cognitively mediated reactions to controlled, cognitively mediated reactions and, across all expression channels, children learn via their social experiences more effective ways to communicate their emotions to others [19, 41]. Although our findings are consistent with the idea of sequential development from vocal to facial and bodily reactions, we acknowledge that our coding system did not pinpoint differing levels of mastery within each expression channel; for instance, our coding system was not designed to differentiate a frustrated grunt from a full syntactical sentence of frustration layered with sarcasm, on anything other than emotional intensity. Therefore, further research is needed to further explore the developmental trajectory of the channels of physiological and communicative emotional concordance in TD children.

Children on the autism spectrum did not differ in their physiological responses to the stress relative to the rest task compared to their TD peers, which is consistent with some reports but not others (for review, see [56]). Contrary to expectations, children on the autism spectrum did not show fewer behaviors overall to express their stress, but rather these were not aligned with their physiological reactions in the way they were in the TD children. This finding suggests that the tendency of children on the autism spectrum to not communicate emotions clearly may specifically be related to expressing internal physiological arousal or stress.

The findings are supportive of a disconnect between internal physiological emotional reactions and external communicative reactions in preschool children on the autism spectrum, such that when children on the autism spectrum are stressed, they do not communicate this in the same way behaviorally as do TD children. This is particularly problematic for the development of emotion regulation, which is socialized throughout development via parental responsiveness to subtle distress cues from their children [19, 20]. Children on the autism spectrum may not be expressing these states to parents and, concomitantly, parents miss opportunities to teach their child strategies for regulating their emotion. Because children’s distress remains unchecked and unregulated, their distress then escalates and culminates in challenging behaviors such as aggression and self-injury (30, 68), setting off a maladaptive distress/expression cycle that is much harder for children and parents to navigate.

The current study does not address the issue of an emotional concordance between physiological reactions and subjective experiences of negative emotion; therefore, it is quite possible that children were aware and cognizant of their emotional states, even if they did not use communication behaviors to express these outwardly to others. Indeed, Keith, Jamieson and Bennettoa [48] found a significant association between self-reported anxiety and autonomic arousal in high-functioning adolescents on the autism spectrum. Interestingly, this association was not found with parent-report of their child’s anxiety, further suggesting that from the outsider’s perspective, individuals on the autism spectrum have impairments in externally expressing their internally experienced stress.

There are practical and therapeutic implications of this study. In a school setting, there can be a buildup of negative emotions internally without communication of distress which can be particularly problematic [83]. Not effectively communicating negative emotion can manifest as aggression, hyperactivity, anxiety, or social isolation [39, 63, 70], and can inhibit academic and social success [35]. Therefore, interventions that target emotional communication and regulation via physiological monitoring particularly noticing mild or rising levels of stress in children on the autism spectrum may be useful. Our previous research suggests that select wearable heart rate trackers are a feasible, valid and reliable means to measure stress in children on the autism spectrum [69]. The recent advances in wearable physiological devices such as wrist-band or chest-strap heart rate trackers present a role for the incorporation of such devices to provide important in-the-moment clinical decision support to caregivers, therapists and teachers. They would be able to monitor real-time physiological stress or arousal of children with ASD in multiple settings. Caregivers, therapists, and teachers may predict challenging behaviors in children on the autism spectrum based on the physiologic data and decide on whether an intervention is needed to de-escalate arousal. This has the potential to be particularly impactful with minimally verbal children on the autism spectrum as it opens up a channel of emotional communication which may not be available otherwise. For more verbally fluent children on the autism spectrum, this technology could help to create learning opportunities in that children may be prompted at times of physiological stress to use their words to communicate their emotions. However, the field of digital health is still emerging and the efficacy of heart rate monitoring for supporting interventions has been yet to be demonstrated empirically. Additionally, given the emotional discordance seen in children on the autism spectrum across age groups in the current study, it will be important to provide psychoeducation to caregivers, therapists and teachers about the potential mismatch between the internal states of children on the autism spectrum and the ways in which they outwardly express their emotions.

Lastly, this study represents another example in which knowledge from autism science can inform affective science/emotion literatures, just as affective science/emotion literatures has informed autism science. For example, data from children on the autism spectrum has helped to elucidate the distinction between cognitive and affective empathy (such that children on the autism spectrum often present with affective but not cognitive empathy; [27]), and knowledge on emotion regulation processes and strategies (e.g., reappraisal vs. suppression; [33]) has informed research on emotion regulation in autism (e.g., [85]). The current study highlights emotional disconcordance seen in children on the autism spectrum and the need to recognize early on that some may not actively communicate physiological distress.

### Limitations

First, whilst the inclusion of an age-matched TD group afforded an understanding of normative emotional concordance in the children, the TD and autism groups were unmatched on cognitive ability. Thus, inclusion of a third group of children matched with the autism group on DQ/IQ would have been ideal, although, as indicated, cognitive ability was not associated with the variables of interest in this study. Second, alexithymia was measured in the school-aged children but not in preschoolers, limiting our understanding of the interaction between alexithymia and emotional concordance in younger children. However, to our knowledge, validated measures of alexithymia are currently unavailable for this age group. Indeed, the alexithymia results should be read with caution as measurement of internal processes by external reporters, such as parents, is inherently limited. Third, as preschoolers aged 2–4 years and school-aged children aged 8–12 years participated in the study, we cannot describe emotional concordance in children aged 5–7 years. Future research should aim to incorporate this age group so that we may better chart the development of emotional concordance. Fourth, the emotions of anger, fear, and sadness were grouped as distress. Although these three emotions share characteristics in physiological reactivity and are all emotions of negative affect, they may invoke varying behavioral expressions. Valuable information may have been lost as a result of this grouping, particularly if the task invoked distinct emotions among the two groups; therefore, emotional concordance for specific negative emotions should be studied in further research. Fifth, given the context of the larger school-age study of wearable heart rate trackers, our measure of physiological arousal (heart rate) was not valence-specific or specific to the sympathetic nervous system that is most implicated in stress responses. However, we argue that some insight on affective arousal is still possible with this measure and can, thus, inform the field given the few studies to date that have focused on this topic. Sixth, as this study combined data across two projects, the heart rate data collection and analysis systems were different, and this may have introduced error in the comparisons across age groups. However, previous research has found good reliability and accuracy of wearable PPG and ECG devices for measuring basic indices of cardiac function, like beats per minute, as compared to wired ECG [12, 23, 43, 55, 74, 91, 95, 104, 107], as have we with the devices used in this study [69]. Nevertheless, future research should aim to compare across age groups with the same physiological systems. Lastly, the study was underpowered to find significant group differences in some analyses reported above, hence we urge readers to consider the effect sizes reported herein.

## Conclusions

The study findings are suggestive of emotional discordance in preschool children on the autism spectrum. These findings support the use of wearable sensors which may be useful to facilitate the development of emotional communication and regulation in preschool children on the autism spectrum.

## Supplementary Information

Supplementary Material 1.

## Data Availability

Data analyzed during this study are included in this published article and its supplementary information files. Full data will be available on ResearchGate.

## Abbreviations

ADOS-2: Autism Diagnostic Observation Schedule, second edition
BPM: Beats per minute
CS: Comparison scores
DQ: Developmental quotient
fMRI: Functional magnetic resonance imaging
IRR: Inter-rater reliability
Lab-TAB: Laboratory Temperament Assessment Battery
TD: Typically developing
