# Supplementary material for: Physiological and communicative emotional disconcordance in children on the autism spectrum
Source: J Neurodev Disord. 2024 Sep 4;16:51. doi: 10.1186/s11689-024-09567-4 (PMC11373183; doi:10.1186/s11689-024-09567-4)
Supplement: Supplementary file 1 — Supplementary Material 1. [file 11689_2024_9567_MOESM1_ESM.docx]

**Supplementary Material**

The following zero order correlation matrices are provided to allow for examination of the associations between IQ/DQ and the variables of interest.

**Supplementary Table 1**

*Zero-Order Correlations between Cognitive Ability and the Variables of Interest: Preschool Group*

|  | DQ | Facial | Vocal | Bodily | Rest M BPM | Stress M BPM | Stress Peak BPM |
| --- | --- | --- | --- | --- | --- | --- | --- |
| **TD** |  |  |  |  |  |  |  |
| DQ |  | .27 | -.16 | .17 | -.13 | .12 | -.13 |
| Facial | .27 |  | .35 | .63** | -.15 | -.06 | .11 |
| Vocal | -.16 | .35 |  | .39 | .19 | .45^#^ | .39 |
| Bodily | .17 | .63** | .39 |  | .12 | .04 | -.08 |
| Resting state heart rate (Mean BPM) | -.13 | -.15 | .19 | .12 |  | .76*** | .18 |
| Stress task heart rate (Mean BPM) | -.12 | -.06 | .45^#^ | .04 | .76*** |  | .39^#^ |
| Stress task heart rate (Peak BPM) | -.13 | .11 | .39 | -.08 | .18 | .39^#^ |  |
| **ASD** |  |  |  |  |  |  |  |
| DQ |  | .22 | .26 | .16 | -.35 | .04 | .01 |
| Facial | .22 |  | .71*** | .66** | .10 | -.10 | .28 |
| Vocal | .26 | .71*** |  | .46* | .02 | -.23 | .21 |
| Bodily | .16 | .66** | .46* |  | .40 | .14 | .22 |
| Resting state heart rate (Mean BPM) | -.35 | .10 | .02 | .40 |  | .57* | .42# |
| Stress task heart rate (Mean BPM) | .04 | -.10 | -.23 | .14 | .57* |  | .48* |
| Stress task heart rate (Peak BPM) | .01 | .28 | .21 | .22 | .42^#^ | .48* |  |

***Note.*** BPM = Beats Per Minute; DQ = Developmental Quotient (Mullen Early Learning Composite score), M = Mean. ^#^*p* < .10, **p* < .05, ***p* < .01, ****p* < .001

**Supplementary Table 2**

*Zero-Order Correlations between Cognitive Ability and the Variables of Interest: School-Age Group*

|  | IQ | Facial | Vocal | Bodily | Rest M BPM | Stress M BPM | Stress Peak BPM | Alexithymia |
| --- | --- | --- | --- | --- | --- | --- | --- | --- |
| **TD** |  |  |  |  |  |  |  |  |
| IQ |  | .10 | .18 | .25 | .22 | .14 | .05 | -.22 |
| Facial | .10 |  | .47* | .68** | .10 | .33 | .53* | -.27 |
| Vocal | .18 | .47* |  | .51** | .23 | .24 | .05 | -.31 |
| Bodily | .25 | .68** | .51** |  | .27 | .42^#^ | .62** | -.35 |
| Resting state heart rate (Mean BPM) | .22 | .10 | .23 | .27 |  | .80*** | .33 | -.00 |
| Stress task heart rate (Mean BPM) | .14 | .33 | .24 | .42^#^ | .80*** |  | .66** | .07 |
| Stress task heart rate (Peak BPM) | .05 | .53* | .05 | .62** | .33 | .66** |  | -.00 |
| Alexithymia | -.22 | -.27 | -.31 | -.35 | -.00 | .07 | -.00 |  |
| **ASD** |  |  |  |  |  |  |  |  |
| IQ |  | -.14 | -.07 | .27 | -.05 | .11 | .15 | .19 |
| Facial | -.14 |  | .00 | .22 | .32 | .06 | .09 | -.35 |
| Vocal | -.07 | .00 |  | .28 | .11 | .28 | .32 | -.19 |
| Bodily | .27 | .22 | .28 |  | .33 | .39^#^ | .39^#^ | -.20 |
| Resting state heart rate (Mean BPM) | -.05 | .32 | .11 | .33 |  | .72*** | .64** | -.00 |
| Stress task heart rate (Mean BPM) | .11 | .06 | .28 | .39^#^ | .72*** |  | .91*** | -.09 |
| Stress task heart rate (Peak BPM) | .15 | .09 | .32 | .39^#^ | .64** | .91*** |  | -.05 |
| Alexithymia | .19 | -.35 | -.19 | -.20 | -.00 | -.09 | -.05 |  |

***Note.*** BPM = Beats Per Minute (heart rate); IQ = Developmental Quotient (Stanford Binet Abbreviated IQ score); M = Mean. ^#^*p* < .10, **p* < .05, ***p* < .01, ****p* < .001

**Supplementary Table 3**

*Correlations between Physiological and Communicative Reactivity in Each Group, Excluding PPG Data in the School-Age Group*

|  | **TD** | | **ASD** | |
| --- | --- | --- | --- | --- |
|  | Heart Rate (BPM) | | | |
|  | Mean | Peak | Mean | Peak |
| **Vocal** | .12 | -.09 | .29 | **.37^#^** |
| **Facial** | **.47*** | **.52*** | -.25 | -.16 |
| **Bodily** | **.41^#^** | **.58*** | .13 | .09 |

*Note.* ASD = Autism Spectrum Disorder group; TD = Typically Developing group. ^#^*p* < .10, **p* < .05, ***p* < .01, ****p* < .001. The pattern of findings shown above was identical to those shown in Table 3 of the manuscript.

**Supplementary Table 4**

*Correlations between Physiological and Communicative Reactivity in Each Group with Raw (Uninterpolated) Data in the Preschool Group*

|  | **TD** | | **ASD** | |
| --- | --- | --- | --- | --- |
|  | Heart Rate (BPM) | | | |
|  | Mean | Peak | Mean | Peak |
| **Vocal** | **.49*** | **.74***** | -.24 | -.01 |
| **Facial** | -.06 | **.40^#^** | -.16 | .14 |
| **Bodily** | -.04 | -.01 | -.11 | .01 |

*Note.* ASD = Autism Spectrum Disorder group; TD = Typically Developing group. ^#^*p* < .10, **p* < .05, ***p* < .01, ****p* < .001. The pattern of findings shown above was identical to those shown in Table 3 of the manuscript.

**Supplementary Table 5**

*Correlations between Alexithymia and Emotional Concordance in Each Diagnostic Group*

|  | **TD** | | | | | | **ASD** | | | | | |
| --- | --- | --- | --- | --- | --- | --- | --- | --- | --- | --- | --- | --- |
|  | BPM & Vocal | | BPM & Facial | | BPM & Bodily | | BPM & Vocal | | BPM & Facial | | BPM & Bodily | |
|  | M | Peak | M | Peak | M | Peak | M | Peak | M | Peak | M | Peak |
| Alexithymia | **-.32^#^** | **-.32^#^** | -.27 | -.27 | **-.37^#^** | **-.36^#^** | -.20 | -.20 | **-.36^#^** | **-.36^#^** | -.21 | -.21 |

*Note.* All correlations are partial, controlling for baseline physiology. Alexithymia = Children’s Alexithymia Measure total score. *^#^p < .10, *p < .05, **p < .01.* After the False Discovery Rate correction applied (Bejamini & Hochberg, 1995), no findings remained significant at *p* < .05.
